# Neuronal Injury and Glial Changes Are Hallmarks of Open Field Blast Exposure in Swine Frontal Lobe

**DOI:** 10.1371/journal.pone.0169239

**Published:** 2017-01-20

**Authors:** Srinivasu Kallakuri, Alok Desai, Ke Feng, Sharvani Tummala, Tal Saif, Chaoyang Chen, Liying Zhang, John M. Cavanaugh, Albert I. King

**Affiliations:** Department of Biomedical Engineering, Wayne State University, Detroit, Michigan, United States of America; Nathan S Kline Institute, UNITED STATES

## Abstract

With the rapid increase in the number of blast induced traumatic brain injuries and associated neuropsychological consequences in veterans returning from the operations in Iraq and Afghanistan, the need to better understand the neuropathological sequelae following exposure to an open field blast exposure is still critical. Although a large body of experimental studies have attempted to address these pathological changes using shock tube models of blast injury, studies directed at understanding changes in a gyrencephalic brain exposed to a true open field blast are limited and thus forms the focus of this study. Anesthetized, male Yucatan swine were subjected to forward facing medium blast overpressure (peak side on overpressure 224–332 kPa; n = 7) or high blast overpressure (peak side on overpressure 350–403 kPa; n = 5) by detonating 3.6 kg of composition-4 charge. Sham animals (n = 5) were subjected to all the conditions without blast exposure. After a 3-day survival period, the brain was harvested and sections from the frontal lobes were processed for histological assessment of neuronal injury and glial reactivity changes. Significant neuronal injury in the form of beta amyloid precursor protein immunoreactive zones in the gray and white matter was observed in the frontal lobe sections from both the blast exposure groups. A significant increase in the number of astrocytes and microglia was also observed in the blast exposed sections compared to sham sections. We postulate that the observed acute injury changes may progress to chronic periods after blast and may contribute to short and long-term neuronal degeneration and glial mediated inflammation.

## Introduction

Operations Iraqi Freedom (OIF) and Enduring Freedom (OEF) have highlighted the emergence of Blast Induced Neurotrauma (BINT) and the associated mild traumatic brain injury (mTBI) as the signature wound in returning service members [1,2]. Shell shock and post-concussive syndrome had a similar prominence during World Wars I and II [3]. With much of these injuries more recently sustained following exposure to an improvised explosive device, basic understanding of the mechanisms and pathological changes in the central nervous system following an open field blast exposure still remains an area of intense research focus. What is still not well studied is whether exposure to primary blast wave causes changes in the gyrencephalic brain. Understanding the pathological changes in the brain following an open field exposure is important considering the complex neurological problems reported in the exposed service members. For example, the history of blast related mTBI has been significantly associated with post-traumatic stress disorder (PTSD) and other physical problems in veterans from OIF [4] as well as those from OEF and Operation New Dawn [5,6]. In addition, the number of exposures also appears to be contributing to the neuropsychological sequelae with increased symptom reporting, as revealed by significant Neurobehavioral Symptom Inventory (NSI) scores in veterans with increased blast exposures [7]. There were also reports of abnormal hormonal levels in one or more pituitary axes [8] in those affected by blast mTBI. Visual dysfunction [9,10] and co-occurrence of auditory, visual and vestibular impairment referred to as multisensory impairment (MSI) was also reported [11] in blast victims. In a sample assessment of veterans, a diagnosis of non-epileptic seizures was suspected in 44% of the samples studied and PTSD was confirmed in 81% of these samples studied. Exposure to blast was considered as the mechanism of the TBI [12] in these samples. In fact, TBI independent of an injury mechanism (blast or non-blast) has been described as a primary driver of adverse outcomes in an analysis of US military personnel [13].

The pathological basis of blast-induced changes is still not well understood. A diffusion tensor imaging analysis of service members revealed that blast mTBI was associated with a pattern of lower white matter integrity, with a larger number of low fractional anisotropy (FA) voxels in those with more than one blast mTBI than in individuals with a single blast injury [14]. Others, albeit using a limited number of blast exposed soldiers also reported abnormalities consistent with cerebellar white matter injury in 3 of 4 subjects studied using diffusion tensor imaging [15]. Compared to control subjects, veterans with blast mTBI also showed neurometabolic changes with significant reductions in the ratio of N-acetylaspartate to choline (NAA/Ch) and N-acetyl aspartate to creatine (NAA/Cr) in the anterior portions of the hippocampus [16].

With limitations in studying brain pathological changes in the blast victims, several animal models using a shock tube system to simulate blast overpressure exposure have gained prominence. Behaviorally, transient anxiety-like behavior in an open field arena was reported in mice subjected to 172–276 kPa blast overpressure with symptoms becoming prominent in those exposed to 345–414 kPa [17]. In rats exposed to blast waves with peak reflected overpressures of either 100 or 450  kPa (39 or 110  kPa incident pressure respectively), FA revealed significant brain abnormalities as evidenced by greater numbers of significant voxels in animals exposed to high-blast compared to low-blast. The decreased FA was observed prominently in the cortex, thalamus and ipsilateral ventral hippocampus [18]. In mice exposed to blast overpressure via a shock tube, Huber et al reported elevated phospho and cleaved-tau species in neurons, as well as elevated manganese superoxide-dismutase levels by 24 hours with the aberrant tau species persisting for at least 30 days post-exposure in the hippocampus [19]. Electrophysiolgically, recordings from the corpus callosum of rat brain slices exposed to blast in the range of 28 kPa indicated greater deficits in unmyelinated fibers relative to myelinated fibers. There was a reduced compound action potential amplitude at 14 days post-injury [20]. Mice subjected to open field explosion (35 and 17 kPa), demonstrated behavioral changes with increased blood brain barrier permeability, increased FA and decreased radial diffusivity that correlated with sites of up-regulation of manganese superoxide dismutase 2 in neurons and CXC-motif chemokine receptor 3 around blood vessels in the fiber tracts [21].

Histologically, in rats subjected to sub-lethal open field blast overpressure (49 kPa or 77 kPa), darkened and shrunken cortical neurons were observed one day after blast with signs of recovery by 4 and 7 days after blast [22]. They also reported increased number of cells with amyloid precursor protein staining in the white matter [22] with some reporting multi focal axonal injury in the cerebellum, corticospinal tract and optic tract in mice subjected to a static blast pressure of 68 kPa using a shock tube [23]. Blast induced swollen neurons and myelin debris in the hippocampus [24] and accumulation of phosphorylated neurofilament-heavy chain (pNF-H) in neuronal perikarya of the cortex attributed to a disturbed axonal transport machinery was also reported [25]. Using silver impregnation technique, investigators have also shown axonal pathology in the cortex and cerebellum of swine exposed to blast pressures of 379 or 538 kPa [26] and in deep cerebellar white matter tracts and various brainstem regions of rats with body shielding exposed to a single 241 kPa blast wave [27]. Others showed cytoskeletal damage in the cortex and hippocampus 7 days after blast exposure using neurofilament immunohistochemistry [28]. With the evidence of axonal injury potentially in the form of impaired axoplasmic transport (IAT) in various blast models, it was reasoned that immunostaining for beta amyloid precursor protein (β-APP) and neurofilament light chain, the markers of traumatic axonal injury [29,30] may offer valuable clues on the extent of neuronal injury in the swine brain following blast exposure.

Glial alterations are another key component of blast induced injury changes in the brain [31]. Alterations related to astrocytes in the brain were prominently shown by several investigators using glial fibrillary acidic protein immunohistochemistry [32–37]. Current research also supports profound microglial activation following blast [38–45] in various animal models. However, studies aimed at understanding changes related to axonal injury and glial proliferation and their quantification in a gyrencephalic brain after a primary blast exposure are limited. Thus, the purpose of this study was to assess neuronal and glial reactivity changes in brains from male Yucatan swine subjected to a single open field blast exposure. Our results from analyses of sections encompassing the frontal lobe of the brain show neuronal and glial injury changes in the gray and white matter regions following open field blast exposure.

## Materials and Methods

### Animal preparation

All procedures were approved by the Institutional Animal Care and Use Committee (IACUC,Wayne State University, Detroit, MI) and the United State Army Medical Research and Materials Command Animal Care and Use Review Office (USAMRMC ACURO). All animals (male Yucatan swine 50–60 kg, 13.4±1.3 months Sinclair Bio Resources LLC, Columbia, MO 65205) were allowed to acclimate to their new housing conditions in the animal quarters prior to any test procedure. On the day of the test, each animal was sedated by an initial intramuscular injection of Ketamine (10 mg/kg once) and xylazine (2 mg/kg once) or ketamine (10 mg/kg once), dexmedetomidine (0.04 mg/kg IM) and acepromazine (0.1 mg/kg IM) in the case of animals subjected to high blast overpressure in the open field blast. Animals subjected to high blast overpressure were also administered buprenorphine sustained release (0.12–0.24 mg/kg;subcutaneous) prior to blast exposure.

Following the initial anesthesia, the animal was intubated and was allowed to breathe spontaneously. Additional ventilatory support was given by an Ambu bag as needed. In animals exposed to medium blast overpressure open blast and sham procedures, anesthesia was maintained by an intravenous infusion of Propofol (12–20 mg/kg). In animals subjected to high blast overpressure, anesthesia was further maintained by intravenous injection of ketamine (5–10 mg/kg/hr) and dexmedetomidine (0.005–0.018 mg/kg). Supplemental intra venous (IV) fluids were administered (Lactated Ringer’s Solution 5–10 ml/kg/hour IV) via an intravenous catheter placed in an ear vein. All the animals were transported in an emergency medical services (EMS) vehicle to a test site (Ares Inc., Port Clinton, OH) for open field blast exposure. After blast exposure, the animals were returned to their housing location and monitored till they recovered from the influence of anesthesia. All animals received trained veterinary care throughout their survival period. A total of 17 animals were tested as part this study. In our experience no animal died prematurely or need to be euthanized during these tests. Based on the experimental design, all animals were allowed to survive for a period of 3 days.

### Animal preparation

All the blast tests were performed on days with no rain or snow. Open field blast overpressure was generated by detonating 3.6 kg of a spherical composition-4 (C4) charge. To attain a single Friedlander waveform, the height of burst was determined to be 0.8–0.9 m and was achieved by suspending the C4 from a metal chain. The animal with an abdominal and thoracic lead shielding (39 kg/sq m) was suspended prone in a canvas harness. The harness was further supported by a steel frame which was suspended from a metal beam (3.7 m off the ground) mounted on two A-frames (S1 Fig). The height of the triple point as a function of the horizontal distance from charge was calculated for the given height of the burst. To expose animal's head below the triple point, the animal was suspended 0.9 m above the ground. The snout of the animal also was supported by two webbing straps to minimize head motion. The eye level was at 0.9 m (3 ft) above the ground with the head facing the direction of the wave propagation (S1 Fig). After proper alignment of the head with respect to the center of the C4 charge, the steel frame was further tied to four hooks cemented to the concrete ground with straps to prevent excessive motion during the blast exposure.

The intensity of the two blast exposures (medium versus high) was achieved by changing the stand-off distance between the animal's head and the center of the C4 charge (Table 1). The actual side-on overpressure was measured by a pencil probe (PCB137A24, PCB Piezotronics, Depew, New York 14043) mounted on a metal frame that was bolted to the ground and placed next to the animal's head at the same height (0.9 m). The overpressure data was acquired at a sampling rate of 1 MHz using the SIRIUS HS-ACC MODULE (DEWESoft, Slovenia). Blast animals were divided into two groups that were exposed either to a single medium blast overpressure (stand-off distance = 3.6 m, n = 7) or high blast overpressure (stand-off distance = 3.1 m, n = 5; S1 Fig). Sham animals (n = 5) were exposed to identical test conditions but were not subjected to blast exposure.

**Table 1:** shows the number of animals studied for each group and the recorded open field blast overpressure.

Sham	Medium Blast Overpressure	High Blast Overpressure
Swine 1	Swine1: 223.5 kPa	Swine1: 359.9 kPa
Swine 2	Swine 2: 332.3 kPa	Swine 2: 359.9 kPa
Swine 3	Swine 3: 305.4 kPa	Swine 3: 403.3 kPa
Swine 4	Swine 4: 222 kPa	Swine 4: 403.3 kPa
Swine 5	Swine 5: 262.7 kPa	Swine 5: 350.3 kPa
	Swine 6: no data	
	Swine 7: 290.3 kPa	

Stable heart rate with no jaw tone, palpebral reflex, and limb withdrawal reflex were used as indicators of adequate depth of anesthesia. Immediately before/during/after blast, SpO2 and heart rate were monitored by pulse oximetry. Body temperature was monitored continuously by a rectal thermometer. At lower ambient temperatures, supplemental heat (water circulating heating pad or Thermacare Heat Wraps) was provided to maintain body temperature.

### Brain tissue processing

All animals were allowed to survive for 3 days after blast or sham procedures. At the end of their survival period, each animal was first injected with Heparin followed by an overdose of sodium pentobarbital and prepared for transcardial perfusion by 4% paraformaldehyde. After ensuring that there was no noxious and palpebral response, the animal was placed supine on a surgical table. The left and right common carotid arteries were exposed and traced inferiorly to the point of their origin in the aortic arch and the connector end of an intravenous tube was inserted into the right common carotid artery and was secured in place by a suture. Each animal was perfused first with 1 liter of saline followed by 8–10 liters of 4% paraformaldehyde. The venous return was collected from the right atrium by a suction pump. The perfusion of lower body was minimized by clamping the aorta just inferior to the heart. Following perfusion, the brain was removed and post-fixed in 4% paraformaldehyde with 20% sucrose until processing for immunohistochemical analyses.

Each harvested brain was cut into 5 mm blocks using a swine brain slicer (Zivic Instruments, Pittsburgh, PA 15237). Each block was further cryoprotected in 30% sucrose and then embedded in an optimum cutting compound. Each block was then further cut into 35–40 μm thick frozen serial sections (S2 Fig) using a cryostat. A total of twenty-four pairs of sections from each of the six blocks were collected in phosphate buffered saline-filled 2 ml vials and stored at 4°C. Then some of the designated sections were probed by various stains to assess cellular injury changes. For each stain, two sections from each block were used. Briefly, the sections were subjected to antigen retrieval by incubation in a citrate buffer (pH 6.0) at 90°C for 1 hour. Then they were immersed for 1 hour in 0.6% hydrogen peroxide to quench endogenous peroxidase activity.

For assessing axonal injury changes, individual sections were subjected to incubation in antibodies against beta amyloid precursor protein (β-APP; 1:250, Cat # 51–2700, Life Technologies, Grand Island, NY), neurofilament light chain (NF-L; 1:5000, Cat#AB9568; Millipore, Temecula CA) or neurofilament-medium chain (NF-M; 1:750; cat # 34–1000, Invitrogen, Camarillo, CA, 1:750). For assessing astrocytic changes, a set of representative sections were incubated in a solution containing antibodies against glial fibrillary acidic protein (GFAP for identifying astrocytes, cat # NE1015, Calbiochem, La Jolla, CA; 1:5000). Microglial activation was detected by incubating a separate set of sections in a solution containing antibodies against ionized calcium-binding adapter molecule 1 (Iba1, cat # 019–19741, Wako Chemicals, Richmond, VA; 1:20000). All the antibody solutions were diluted in 2% normal goat serum (Vector Laboratories, Burlingame, CA) mixed in 1% bovine serum albumin (BSA). After respective primary antibody incubation, sections were incubated in a solution containing a 1:500 dilution of respective biotinylated secondary antibody (Vector Laboratories, Burlingame, CA) followed by exposure to Vectastain Elite ABC reagent. Finally, the peroxidase activity was developed by brief incubation in 3, 3’-diaminobenzidine and hydrogen peroxide. The sections were washed, dehydrated and cover slipped using DPX (Sigma Aldrich). In control incubations, normal goat serum was substituted for primary antibody.

#### Quantification of β-APP immunoreactivity

The presence of neuronal injury as evidenced by β-APP reactive zones was quantified from one section of each block from sham (n = 5 animals x 6 sections per animal = 30 total sections), medium blast overpressure (n = 7 animals x 6 sections per animal = 42 total sections) and high blast overpressure (n = 5 animals x 6 sections = 30 total sections) groups. In the gray matter, an immunoreactive zone was defined as the region encompassing intense β-APP-reactive cell bodies that was observed in various cortical layers including the molecular layer. In the white matter, immunoreactive zones were defined as aggregates of intense β-APP-reactive axon-like processes, individual retraction balls, axonal swellings or intensely stained stellate-like cells. For quantification, each section (encompassing the entire left and right hemispheres) was viewed under a light microscope (Leica DMLB, Leica Microsystems Ltd, Heerburg, Switzerland) at 50x magnification defined as the high power field. The presence or absence of β-APP immunoreactive zones in these high power fields was investigated by a blinded investigator and the total number of gray and white matter immunoreactive zones for each section was recorded.

#### Quantification of astrocytic and microglial reactivity

Quantification of astrocytes and microglia was performed using separate sets of sections stained for GFAP and Iba1 respectively. For astrocytic quantification in a blinded fashion in blast and sham groups, a x5 composite panoramic image from two sections each per block (sham n = 60 sections; medium blast overpressure n = 84 sections; high blast overpressure n = 60 sections) was taken using OASIS software (Objective Imaging Inc., Kansasville, WI 53139). These panoramic images were then used as a guide to obtain a series of five x100 images per hemisphere, which in turn were used exclusively for counting the number of astrocytes. As the GFAP staining was predominantly found in the white matter tracts, representative images spaced approximately 5 mm apart encompassing the white matter regions were obtained. Then, the number of astrocytes in each such image (10 images per section; 120 images for six blocks per animal resulting in a total of 2040 images for all the 17 animals) were counted.

For quantifying microglia in blast and sham groups another x5 composite panoramic image from two sections each per block per stain (sham n = 60 sections; medium blast overpressure n = 84 sections; high blast overpressure n = 60 sections) was taken using OASIS software (Objective Imaging Inc., Kansasville, WI 53139). However, as microglia were observed in both the white and gray matter regions, using the x5 panoramic images as a guide, another set of five representative images at x100 magnification encompassing areas of both the white and gray matter were taken per hemisphere. This resulted in a total of (10 images per section; 120 images for six blocks per animal) 2040 images for all the 17 animals exclusively for counting microglia. The number of astrocytes and microglia were quantified independently from their respective images using ImageJ. Each image was inverted using the invert option which results in a reverse image similar to the negative of a photograph and enables better delineation of cells of interest from their background.

The average number of β-APP immunoreactive zones in gray and white matter, astrocytes and microglia per group was calculated and compared statistically for group-wise differences using Statistical Package for the Social Sciences (SPSS, IBM) software. One-way analysis of variance (ANOVA) was used for comparisons across study groups. Post hoc LSD tests were used for pair-wise comparisons as appropriate. A probability level of *p* <0.05 was considered statistically significant.

### Serum analyses for injury markers

Besides collecting the brain for histological analyses, serum samples were collected before and at 6 hours, 24 hours, 48 hours and 72 hours after the blast or sham procedures. These samples were assessed for temporal changes in the expression of phosphorylated neurofilament heavy chain (pNF-H Elisa Kit, Encor Biotechnology, Gainesville, FL), glial fibrillary acidic protein (GFAP; cat#NS830, EMD Millipore, Billerica, MA) and interleukin-6 (IL-6; cat# ESIL6, Life Technologies, Carlsbad, CA) by enzyme-linked immunosorbent assay (ELISA) as per the manufacturer's instructions. The ELISA data were analyzed by generalized estimating equations (GEE) with an exchangeable correlation structure and robust standard errors. Baseline biomarker data were entered as a covariate to address possible baseline imbalance.

## Results

### Axonal injury: β-APP immunohistochemistry

**Fig 1:**
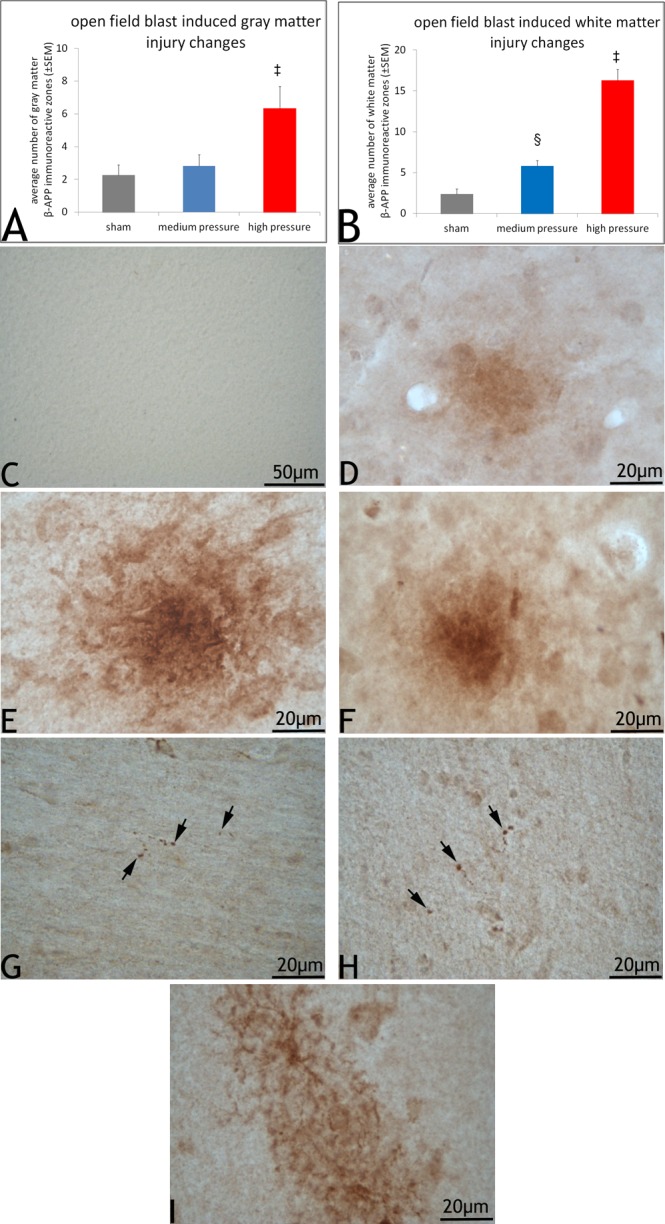
β-APP immunoreactivity in the blast and sham swine. 1A shows average number (± standard error of the mean, SEM) of β-APP immunoreactive zones in the gray matter of blast and sham swine sections. The number of β-APP immunoreactive zones in the gray matter of high blast overpressure sections were significantly high (‡) compared to those in sham and medium blast overpressure (*p<0*.*05*; LSD; One-way ANOVA). 1B shows average number (± standard error of the mean, SEM) of β-APP immunoreactive zones in the white matter of blast and sham swine sections. The number of β-APP immunoreactive zones in the white matter of high blast overpressure sections (‡) were significantly high compared to those in sham and medium blast overpressure (*p<0*.*05*; LSD; One-way ANOVA). Furthermore, the extent of white matter immunoreactive zones was also significantly high in the medium blast overpressure sections (§) compared to sham (*p<0*.*05;* LSD; One-way ANOVA). 1C shows a control section stained without the primary antibody showing no apparent immunoreaction. 1D shows a sham section showing a less intense gray matter β-APP immunoreactive region. 1E and 1F shows an intensely stained gray matter immunoreactive zone from a representative high and medium blast overpressure swine sections. 1G and 1H show representative β-APP reactive retraction balls in the cortical white matter tracts. 1I shows an immunoreactive zone with stellate-like profiles in the white matter tracts of a swine subjected to high blast overpressure.

Our investigation revealed more prominent β-APP immunoreactive zones in the open field blast exposure group than in the sham group. A further analysis revealed that the number of β-APP immunoreactive zones in the gray matter of high blast overpressure group was significantly high compared to those from both the medium blast overpressure and sham groups (*p<0*.*05)* with no other changes (Fig 1A). Immunoreactive zones in the gray matter were observed in various cortical layers characterized by 1–3 intensely stained cell bodies with plaque-like deposits with extended processes of the cell bodies (Fig 1E and 1F). These immunoreactive zones were also observed in the sub-pial regions of the cortex. The components of β-APP immunoreactive zones in the white matter regions were the presence of axons undergoing pathological changes. A further analysis of these white matter injuries revealed that the extent of these injuries in the high blast overpressure group was significantly high compared to the medium blast over pressure and sham groups (*p<0*.*05)*. Furthermore, the extent of these injuries was also significantly high in the medium blast overpressure group compared to sham (*p<0*.*05;* Fig 1B). These pathological changes were in the form of single or multiple axonal swellings, swollen axons with terminal retraction balls and retraction balls with or without tail-like profiles (Fig 1G and 1H). These axonal injury profiles were particularly predominant in the sub-cortical coronal radiations ending in various sulci, more so in the dorsal aspect of the cerebral hemispheres. Other areas that also showed injured axons were internal capsule, white matter bundles of the striatum, and white matter fibers near the vomiro nasal organ. Occasionally, axonal injury was also observed in the periventricular white matter tracts and corpus callosum. Immunoreactive zones in the white matter were also associated with stellate-like cells with their dark stained processes surrounded by retraction balls and axons showing signs of swellings (Fig 1I). Immunoreactive zones in sham sections tended to be less intense (Fig 1D) with no staining in control sections (1C).

### Axonal injury: NF-L and NF-M immunohistochemistry

**Fig 2:**
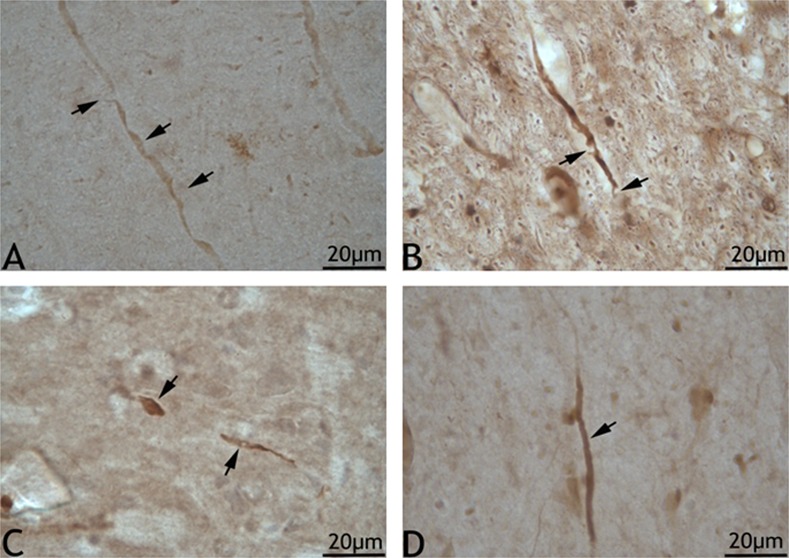
NF-L immunoreactive large caliber nerve fibers in blast and sham sections. 2A shows an axon with swollen regions (arrows) in a brain section from the medium blast overpressure group. 2B and 2C show swollen axons with apparent vacuolations and retraction balls in a section from the high blast overpressure group. 2D shows a normal appearing axon in a section from the sham group.

**Fig 3:**
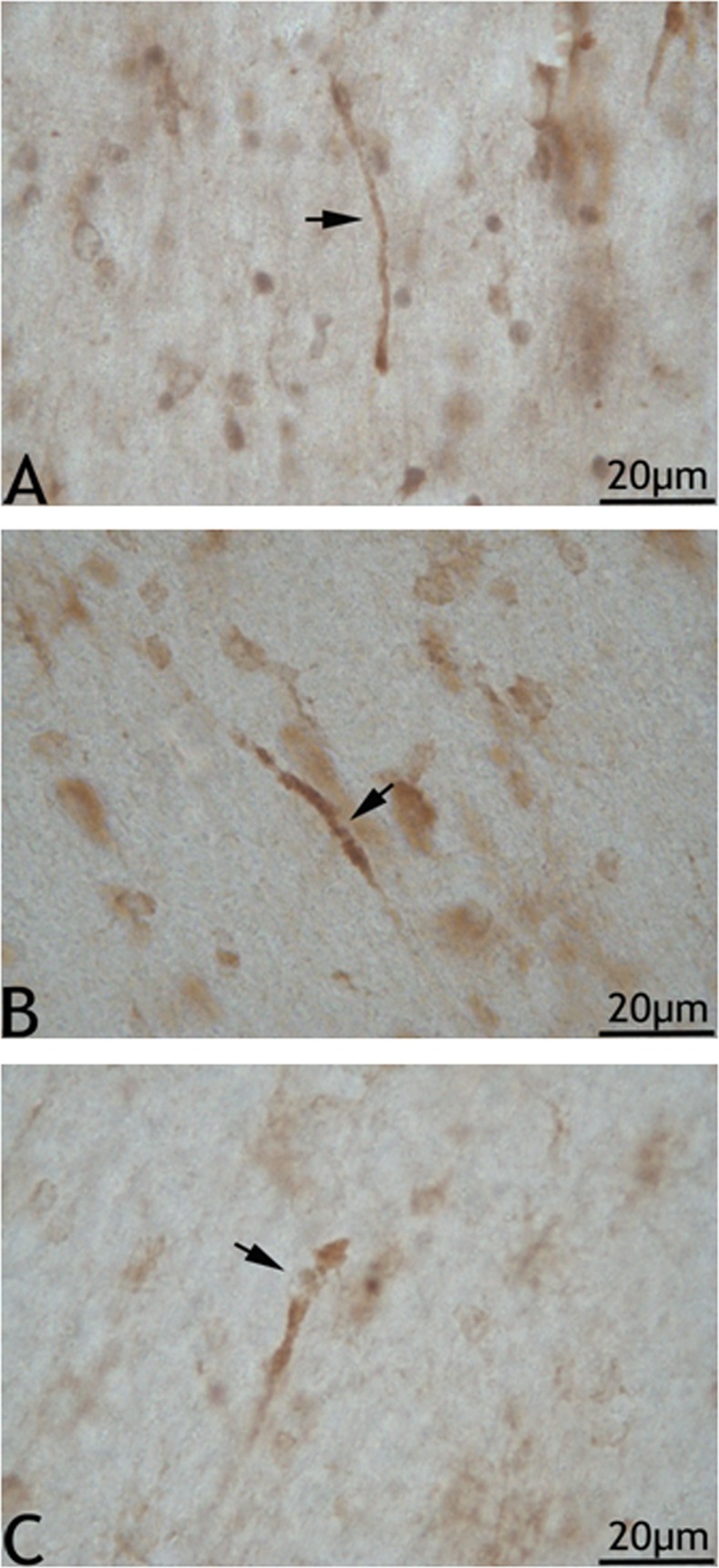
NF-M immunoreactive large caliber axons in the cortical radiations. 3A shows normal looking axon in a sham brain section. 3B and 3C show apparently reactive axons. 3B shows an axon with an apparent vacuole like (arrow) presence in a section from medium blast overpressure group. 3C shows a large caliber axon with a terminal putative enlargement preceded by vacuolated appearance (arrow) in a section from a high blast overpressure group.

A limited attempt to assess axonal injury by NF-L and NF-M immunohistochemistry was pefomed. In brain sections from swine exposed to medium and high blast overpressure, many NF-L reactive axons with uniform caliber and well-stained axonal core were observed. However, some large caliber axons with altered morphology were also observed (Fig 2A–2C). These axons tended to have membrane boundaries that appeared to be disrupted in the form of semilunar empty space or occasionally with filamentous projections originating from their membranes. Besides, some of the large caliber axons appeared to be swollen and at times with the presence of vacuoles in their axonal core. In addition, some axons with terminal retraction bulbs appearing as clubs were also observed. The location of these observed changes was predominantly in the sub-cortical white matter tracts. In the corpus callosum, no such prominent observations could be made considering the predominant nature of small fiber axons in its population. Qualitatively, in sections from sham animals, NF-L reactive axons running for extended lengths in the white matter tracts were observed. These axons appeared to have uniform caliber with well stained NF-L reactive core (Fig 2D). Axonal injury changes assessed by NF-M immunohistochemistry revealed putative axons that appeared to be linear with occasional swollen regions (arrow) and vacuolations (Fig 3). These limited findings need to be further validated.

### Astrocytic reactivity changes

**Fig 4:**
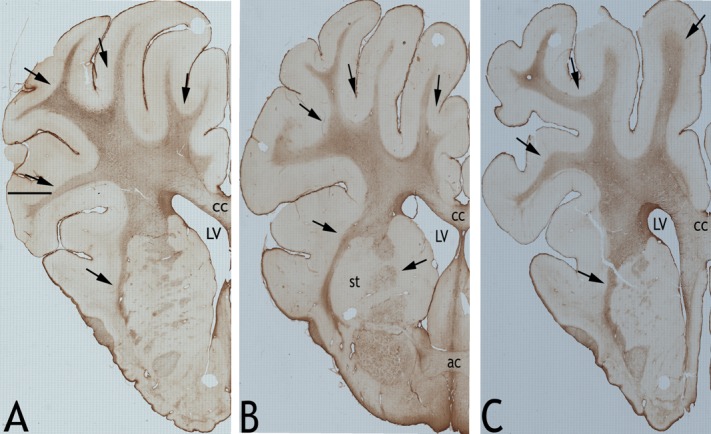
Astrocytic reactivity in the white matter tracts. Representative panoramic images of sections of GFAP stained left hemispheres from a typical sham (A), medium (B) and high (C) blast overpressure exposed swine. Arrows indicate white matter radiations into the cortex and other tracts that appeared to be clearly delineated from the gray matter. Note the intense staining of the neuropile. (CC = corpus callosum; LV = lateral ventricle; st = striatum; ac = anterior commissure).

**Fig 5:**
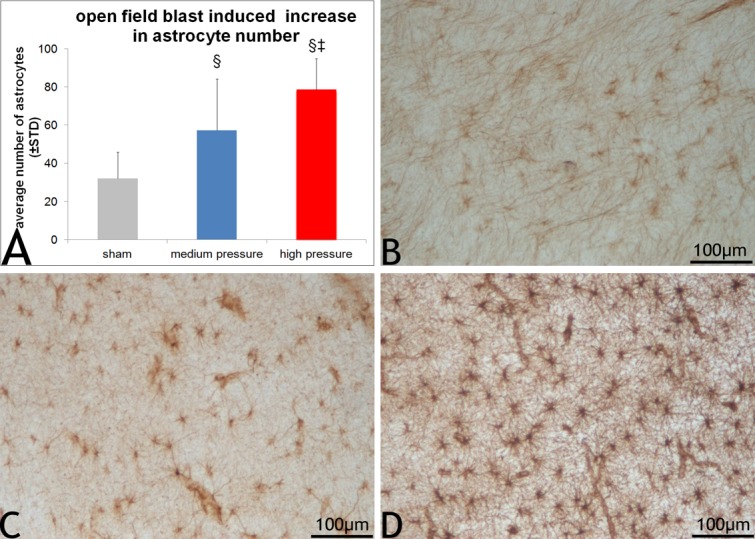
Quantification of open field blast induced astrogliosis. 5A shows the astrocyte counts in the blast and sham groups. Astrocyte counts in sections from the high and medium blast overpressure group were significantly elevated compared to those from sham group. Astrocyte counts in the high blast overpressure group were significantly higher than in the medium blast overpressure group. 5B shows an image of lightly stained astrocytes in a representative sham section. 5C and 5D show increased astrocytic proliferation in medium and high blast overpressure exposed sections. Furthermore, astrocytes in blast sections appeared to be intensely stained with enlarged cell bodies and processes (5D).

In all the sections from the six blocks of the frontal lobes, the astrocytes were almost exclusively localized to the white matter tracts (Fig 4) and thus images were exclusively obtained from the white matter tracts for the purpose of quantification. Although astrocytes in other areas of the cortex and sub-pial regions were observed they were less prominent than in white matter tracts. Quantification of images revealed a significantly high number (*p*<0.05; Fig 5A) of GFAP reactive astrocytes in sections from both the medium and high blast overpressure groups compared to those from sham (Fig 5B). Furthermore, astrocyte counts in sections from high blast overpressure group were also significantly high compared to those from medium blast overpressure (*p<0*.*05)*. Astrocytes in the blast group tended to be intensely stained by GFAP with numerous processes (Fig 5C and 5D). In addition, astrocytes in the blast groups appeared to undergo morphological changes with enlarged cell bodies (Fig 5D; S3 Fig).

### Microglial reactivity changes

**Fig 6:**
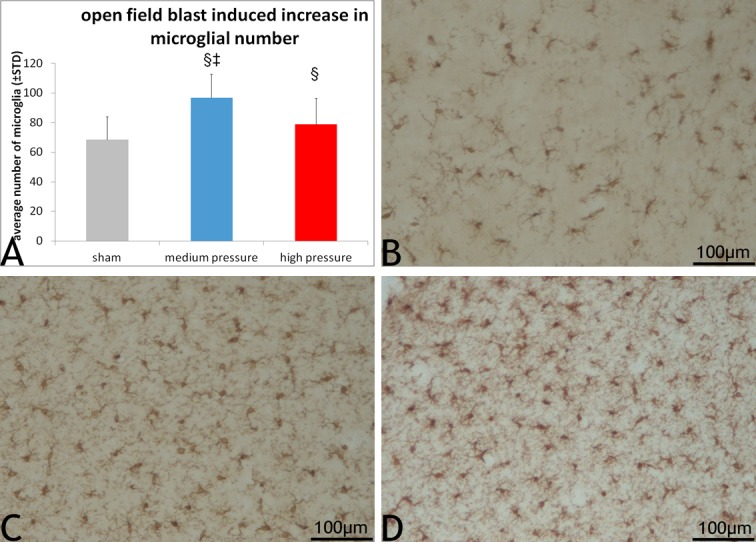
Open field blast induced microglial proliferation. 6A shows extent of the microglial counts in the blast and sham groups. Microglial counts in sections from the high and medium blast overpressure blast groups were significantly high compared to those from sham group. 6B-D show representative images from sham, medium and high blast overpressure exposed sections highlighting variations in microglial proliferation. Microglia in blast sections appeared to be enlarged in size.

In the case of microglia, they were observed throughout the layers of the cortex, sub cortical white matter tracts and other structures with no specific distribution pattern. The number of microglia in both the blast groups was higher than in sections from sham group (*p*<0.05; Fig 6A). Unlike astrocytes, the microglial counts in the medium blast overpressure blast group were significantly higher than in the high blast overpressure group (*p<0*.*05)*. Compared to sham (Fig 6B), microglia in the blast group (Fig 6C and 6D) tended to be more oval in shape with limited number of processes.

### Serum biomarker changes

ELISA data from the three markers was compared for statistical significance using generalized estimating equations. Baseline biomarker data were entered as a covariate to address possible baseline imbalance. Of the three biomarkers analyzed, there was some evidence of higher glial fibrillary acidic protein levels in the high blast overpressure group than the sham group (*p = 0*.*08*). No apparent difference in glial fibrillary acidic protein levels was found between sham and medium blast overpressure group. No significant changes were observed with regard to the serum levels of phosphorylated neurofilament heavy chain and interleukin 6.

## Discussion

To our knowledge, this study is the first of its kind to attempt to address the fundamental question whether an open field blast exposure causes injurious changes in the gyrencephalic brain. Although there were other studies that attempted to address the same question in a gyrencephalic model using explosives, animals in those studies were exposed to a simulated open field blast by positioning the animal either in a shock tube, high mobility multipurpose wheeled vehicle surrogate or in a four-sided building with no roof using a moderate charge [26,37,46]. Another previous open field blast study (2.1 kg explosive) positioned the animal on a steel shelf mounted to the concrete wall of the bunker and studied only physiological parameters such as respiration, circulation and cortical activity but no histological analyses of brain for injury changes as in the current study [47]. Säljö et al on the other hand, offers some details on the effects of repetitive blast pressure (3 times during a 10–15 minute period) in swine exposed to low level noise produced by various weapons (a howitzer, a bazooka, an automatic rifle) or underwater explosives [48]. They reported that animals exposed to bazooka (Pmax 42 kPa) and automatic rifle (Pmax 23 kPa) showed significant increase in subarachnoidal and small parenchymal bleedings in cortical regions with occipital lobe and cerebellum being the most predominantly affected structures. Animals exposed to howitzer blasts at 30 kPa although displayed parenchymal and subarachnoid hemorrhages, they were not significantly different from that of controls due to the limitations in the number of animals. Säljö et al concluded that low levels of blast causes brain edema as indicated by increased bioelectric impedance, an increase in intracranial pressure, small brain hemorrhages and impaired cognitive function [49]. In our study the animals were exposed to higher open field blast pressure than these animals and the likelihood of such hemorrhages although possible was not investigated as the focus was to study neuronal injury and glial reactivity changes.

We studied injury changes in the brain following an open field blast in Yucatan swine suspended in a sling and positioned below the triple point and exposed to a single Friedlander wave form either at medium (range 222 kPa—333 kPa; average 272±5 kPa) or high blast overpressure (range 350–403 kPa; average 375±3 kPa). Our lowest medium blast overpressure of 222 kPa was similar to the mean shock tube blast pressure (241±8 kPa) reported by de Lanerolle et al (2011) in Yorkshire swine. Besides, the shock tube pressures reported by de Lanerolle et al ranged from 131–365 kPa [37] with their lowest pressure range far lower than in our study. In our experience there were no mortalities in both the medium and high blast overpressure groups. These pressures were very close to those utilized by de Lanerolle et al (2011) who reported shock tube and vehicular blast pressures in the range of 255–365 kPa with potentially long durations that may be a contributing factor for the observed mortality [37]. Furthermore, the medium and high blast overpressures used in our study are higher than those used by Gyorgy et al (2011) who used three different blast overpressures of <152 kPa, 138–276 kPa and >276 kPa respectively on Yorkshire swine and reported time dependent changes in serum biomarkers [50].

This initial report offers data on neural, astrocytic and microglial changes from the most anterior aspect of the brain to about 30 mm posterior. Our findings suggest, that open field blast exposure causes neuronal injury and marked increase in the number of astrocytes and microglia as early as three days after blast in the cortical gray and white matter regions of the frontal lobe. Although not investigated, it is plausible that these injury changes can progressively evolve and can extend to more chronic time periods as supported by the findings from de Lanerolle et al who studied changes two weeks after blast in swine [37]. Neuronal injury in the form of beta amyloid immunoreactive cell bodies, stellate cells, axonal swellings and retraction balls in sections from blast group was observed. The axonal injury in the form of terminal retraction balls and beaded profiles was very similar to previous findings of diffuse axonal injury following traumatic brain injury induced by an impact acceleration device [51]. Furthermore, the microscopic nature of these changes may not allow them to be detected either by routine or advanced radiological assessments and may render diagnosis of blast related pathology even more difficult. The immunoreactive zones in gray matter may suggest accumulation of β-APP in the cell bodies that may be related to impaired axoplasmic transport. An increased cytoplasmic β-APP staining in the perikarya following traumatic brain injury also was previously reported [52,53]. Additionally, up-regulation of β-APP in cells including Purkinje cells and hippocampal neurons has been reported in a rodent model of cranial blast [54]. Furthermore, some of the beta amyloid stained regions in the white matter resembled glial cells with projections. For that matter, there is an increase in the number of microglia in both the gray and white matter regions of sections from the blast exposure groups compared to sham. In fact, Ryu et al recently reported APP positive axonal abnormalities in several brain sites from veterans that suffered blast injury that appeared as clusters of axonal spheroids or varicosities in a honeycomb pattern with perivascular distribution in the medial dorsal frontal white matter [55]. The honeycomb appearance of immunoreactivity in their study may be related to the presence of staining around microglial cells that may be responsible for the characteristic honeycomb appearance in the white matter tracts and may need to be validated by dual labeling immunofluorescence for β-APP and microglial markers. β-APP reactive axonal profiles in the medium and high blast overpressure group were observed in sections encompassing the prefrontal and frontal lobes predominantly in the dorsal sub cortical white matter structures. These frontal lobe axonal changes were further supported by NF-L and NF-M immunoreactive axons. Axonal changes evidenced by NF-L immunoreactive swollen profiles, with vacuolations and club-like terminations were observed in various sub cortical white matter tracts. In fact NF-L immunohistochemistry was previously used to show the pathogenesis of diffuse axonal injury following traumatic brain injury [56] with others showing cell body changes evidenced by accumulation of phosphorylated neurofilament heavy chain in rats exposed to explosive blast in a shock tube [25]. Although this needs further validation, we also show some putative signs of neurofilament compaction. Neurofilament compaction is one of the components of traumatic axonal injury [57] with the other being impaired axoplasmic transport revealed by β-APP immunohistochemistry [51,57]. In fact, the predominance of β-APP reactive profiles in the blast groups supports the dominance of impaired axoplasmic transport which was reported to be localized to the thin caliber axons [58] as revealed by very thin β-APP reactive profiles especially in the sub cortical white matter. In addition, the utility of β-APP immunohistochemistry was also reported by other investigators studying blast related changes in rodents [27,59].

The presence of a high number of GFAP reactive astrocytes delineating the white matter in the blast swine brain sections is another hallmark of this study with the number of astrocytes increasing with increasing pressure. The presence of astrocytes delineating the white matter tracts implicates and supports their strong association with ongoing neuronal injury changes in the white matter tracts and to our knowledge this type of white matter delineation was never reported in a gyrencephalic blast brain injury model. De Lanerolle et al also reported astrocytes in subpial cortex and white matter of Yorkshire swine, an observation similar to our findings. However, quantitatively, they reported significant astrocyte increase in regions of hippocampus in swine exposed to blast in a vehicle and building but not in the shock tube setting [37]. On the other hand, Bauman et al using two Yorkshire swine exposed to moderate peak pressure in a high mobility multipurpose wheeled vehicle (HMMVEE) showed astrocytosis in the ipsilateral superior corona radiata (exposed side) of the posterior frontal cortex as well as in multiple layers of the dentate gyrus. They also showed elevated GFAP levels in the ipsilateral frontal cortices of the two swine tested [26]. With the very limited swine studies, much of the knowledge related to blast induced astroglial changes comes from several rodent blast studies [22,32,35,43,60–62]. For example, Svetlov et al showed peak GFAP accumulation in the hippocampus seven days after blast exposure to 358 kPa that persisted for 30 days post-blast by western blot analysis with no significant accumulation in the cortex at any of the survival periods. They also showed significant serum and cerebrospinal GFAP levels at 24 hours and four days after blast respectively [32]. In a separate study that subjected rats either to composite or primary blast, prominent astrocytosis in the hippocampus was reported at 1 and 7 days after blast with elevated serum GFAP levels at 6 hours, 1 day and 7 days respectively following composite or primary blast [35]. Sajja et al using an established shock tube blast overpressure model, reported elevated GFAP levels 24 hours after exposure (117 kPa) but not after 48 hours [60]. Garman et al on the other hand, reported no prominent GFAP and Iba1 staining (although small number of reactive microglia was found in areas of neuronal death) in any of the survival periods studied (24 hours, 72 hours, 2 weeks) in rats subjected to head only exposed blast overpressure of 241 kPa [43]. Turner et al, using a tabletop shock tube, reported graded astrocytic reactivity in the corpus callosum based on the exposed peak overpressure [61] similar to our findings that showed an increased astrocyte count with increasing blast pressure. The histological observation of prominent GFAP in our study is further supported by high GFAP serum levels albeit insignificant in the high pressure group. Taken together, these findings may support GFAP to be a candidate biomarker of blast induced neurotrauma. In a study of Yorkshire pigs, Gyorgy et al did not report injury changes by histology, but showed a time dependent increase in S100B and also reported high variability among animals [50]. Sevtlov et al showed a significant increase in blood GFAP levels by 24 hours with levels in CSF showing a decline and accumulation in a time dependent manner in rats [32].

Another striking observation from our study is the significant increase in the number of microglia in the medium blast overpressure swine sections compared to high blast overpressure and sham groups. This type of differential expression of microglia and astrocytes in swine blast has never been reported. In our study, microglia were observed in both the cortical and white matter regions with no preferential localization. However, de Lanerolle et al although reported no difference in the distribution of microglia in the superior frontal cortex and hippocampus between blast and controls, but observed prominently activated microglia in the central white matter and corpus callosum with no additional quantitative data in swine that survived for 2 weeks after blast [37]. The extent of microglial response at chronic time periods after open field blast is yet to be fully studied. Similar to astrocytes, much of the knowledge related to blast induced microglial changes comes from rodent studies using blast wave [38,41] and impulse noise [39]. Sajja et al in their recent report on pathological changes after low (69 kPa), moderate (97 kPa) and high (165 kPa) shock tube overpressures reported a differential expression of microglia and astrocytes seven-days post-blast survival period, a finding very similar to our observations. They reported significant increase in microglial reactivity in low pressure group alone with increase in astrocytes with increasing pressure [63]. This is different from Turner et al who reported an increase in the number of corpus callosum microglia with increasing pressure (217, 350, and 497 kPa) [36] with Garman et al previously reporting only slight evidence for microglial activation in rats subjected to 241 kPa blast overpressure [27]. On the other hand, Kaur et al reported microglial cells in close association with some darkened dendrites in rats subjected to single non-penetrating blast [38]. A putative pathological implication for microglia comes from studies by Kane et al that showed an increased expression of microglial genes related to immune function and inflammatory responses in cultured microglia subjected to overpressure [44]. Despite several important findings, our study is marked by certain limitations such as lack of functional assessment of the animals following blast exposure and unbiased stereology in histological assessment. Although there was evidence of increasing serum biomarkers levels in the blast groups compared to sham, no significant differences were observed which may be related to wide variations and limited samples size.

What are the potential implications of the observed astrocytic and microglial activation in the frontal lobes of the brain? One implication is their potential role in neurodegeneration. It was recently shown that transforming growth factor beta from immature astrocytes could initiate synaptic elimination in post-natal thalamus by regulating the expression of C1q, a subcomponent of C1 complex of complement activation in the retinal ganglion cells [64]. C1q can trigger classic complement pathway that can lead to tagging of the supernumerary synapses with C3b fragment derived from complement activation and their ultimate elimination by microglia [65,66]. There may also be a bidirectional relationship between the activation of astrocytes and that of microglia. For example, the attenuation of reactive gliosis in a model of AD led to a high number of microglia in the vicinity of plaques and cortex [67]. Whereas the attenuation of astrocytic activation in a mouse model of Batten disease was shown to be accompanied by increased number of microglia in the brain [68]. What could be the potential implication of differential astrocytic and microglial response as observed in this study? Does it mean that at lower pressures, cellular injury changes may be more discrete and modulated by microglia with high pressures leading to activation of both microglia and astrocytes with the latter playing a dominant role? It is very likely that astrocytes may serve as markers of injury severity. As their number increases with increasing pressure, there may be an array of neuronal and inflammatory injury changes as well as potential blood brain barrier permeability disruptions as indicated by their apparent high serum concentrations. Furthermore, the presence of astrocytes almost exclusively delineating white matter tracts may be an indication of the extensive white matter injury following blast exposure. In addition, it is also possible that mechanical perturbations in the tissue trigger astrocyte derived adenosine triphosphate release which may lead to rapid recruitment of microglia to the site of injury and can lead to both microglial and astrocytic reactivity responses [69]. The role of microglia and astrocytes contributing to the release of various inflammatory mediators has been well reported. For that matter, studies by Bauman et al and others have shown increased levels of tumor necrosis factor, interleukin 1 beta, and interferon-γ in cerebrospinal fluid and serum following blast [26,35] lending support also to the inflammatory nature of blast pathology.

In conclusion, our investigation of a gyrencephalic brain three days after open field blast exposure supports the presence of a robust neuronal injury accompanied by extensive astrocytic and microglial activation in the frontal lobes. The severity of the observed neuronal and astrocytic changes appeared to be proportional to the level of blast exposure. The microglial response appears to be differential with high numbers at medium blast overpressure and low numbers at high blast pressure. Whether these injury changes extend to more posterior aspects and brainstem parts is a major aspect of our ongoing investigations. The functional implication of these observed changes may be related to neuronal, axonal and dendritic degeneration combined with a cascade of inflammatory mediator release.

## Supporting Information

S1 Fig Open field blast set-up. Figure shows swine suspended in slings in preparation for open field blast exposure. Red arrows point to two of the three pencil probes positioned to measure the incident pressure at the same standoff distance and height above the ground as the animal head. In this representative set-up, the swine with asterisk was the designated non-instrumented swine used for histological analyses. The other instrumented swine was used as part of a separate investigation to assess the brain biomechanical responses following open field blast exposure.
(TIF)

S2 Fig Methodology of swine brain sectioning. S2A shows the brain slicer with a brain positioned and with blades inserted to harvest a 5 mm block shown in S2B. The arrow in S2B points to the hole made by inserting the tip of a glass pipette to identify the left hemisphere. S2C shows a series of representative blocks obtained from the anterior aspect of a swine brain. Each block was further sectioned into 35–40 μm thick sections. These blocks originate at the most anterior aspect of the frontal lobe and extend 30 mm posterior encompassing the corpus callosum, striatum, internal capsule, lateral ventricles and the septum.
(TIF)

S3 Fig Additional images showing astroglial changes. Astrocytes from sham (A), medium (B) and high blast overpressure (C) exposed groups.
(TIF)
