# Supplementary figures and images for: Neuronal Injury and Glial Changes Are Hallmarks of Open Field Blast Exposure in Swine Frontal Lobe
Source: PLoS One. 2017 Jan 20;12(1):e0169239. doi: 10.1371/journal.pone.0169239 (PMC5249202; doi:10.1371/journal.pone.0169239)

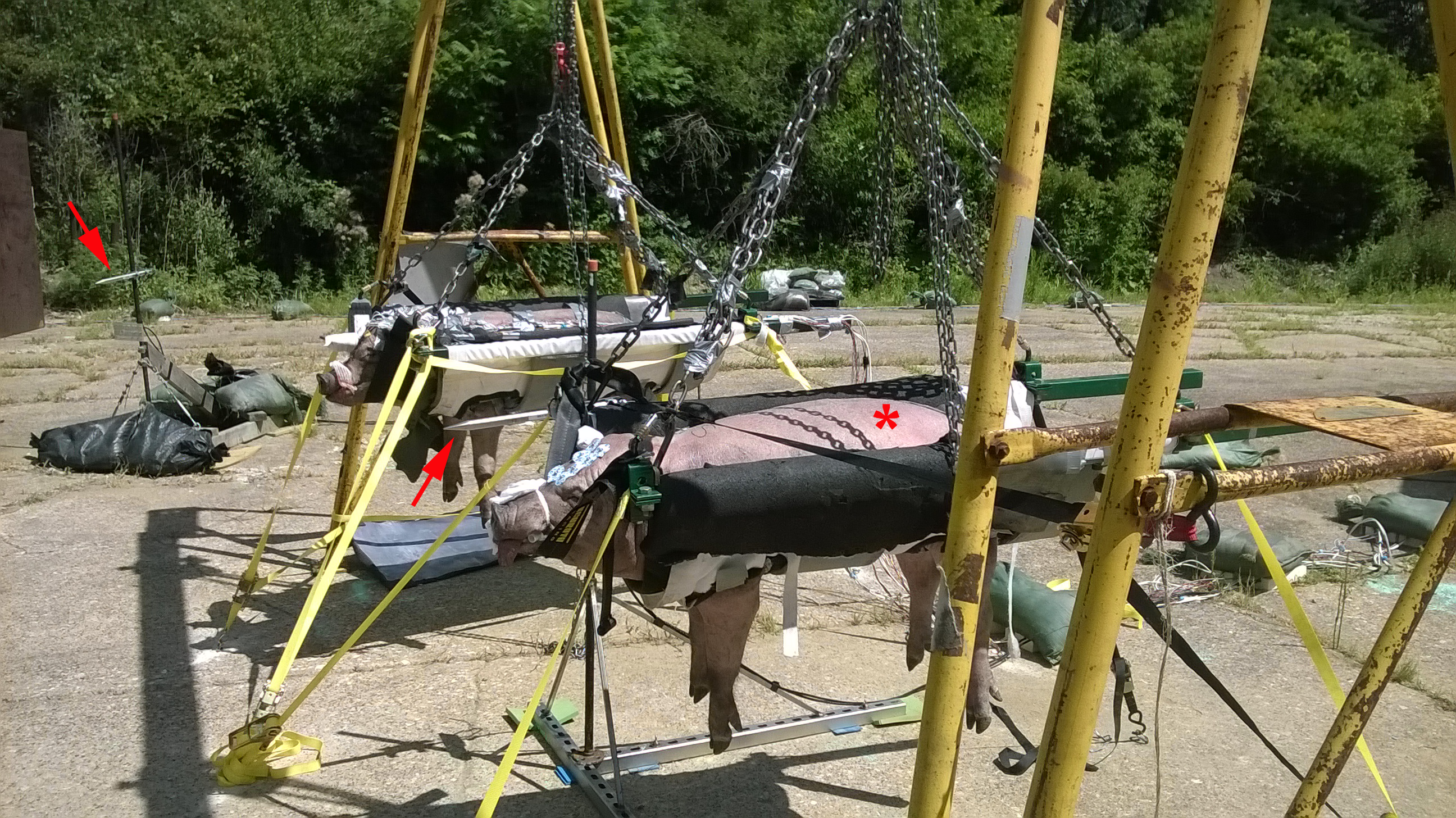

Supplement: S1 Fig — Figure shows swine suspended in slings in preparation for open field blast exposure. Red arrows point to two of the three pencil probes positioned to measure the incident pressure at the same standoff distance and height above the ground as the animal head. In this representative set-up, the swine with asterisk was the designated non-instrumented swine used for histological analyses. The other instrumented swine was used as part of a separate investigation to assess the brain biomechanical responses following open field blast exposure. (TIF) [file pone.0169239.s001.tif]

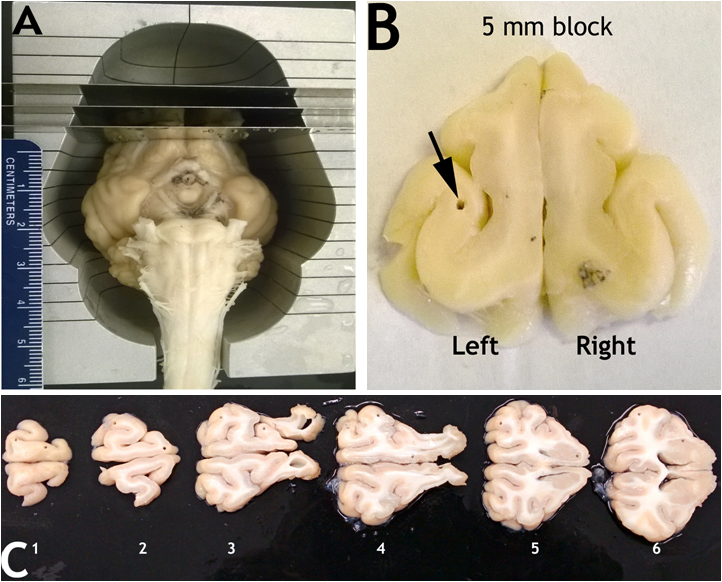

Supplement: S2 Fig — S2A shows the brain slicer with a brain positioned and with blades inserted to harvest a 5 mm block shown in S2B. The arrow in S2B points to the hole made by inserting the tip of a glass pipette to identify the left hemisphere. S2C shows a series of representative blocks obtained from the anterior aspect of a swine brain. Each block was further sectioned into 35–40 μm thick sections. These blocks originate at the most anterior aspect of the frontal lobe and extend 30 mm posterior encompassing the corpus callosum, striatum, internal capsule, lateral ventricles and the septum. (TIF) [file pone.0169239.s002.tif]

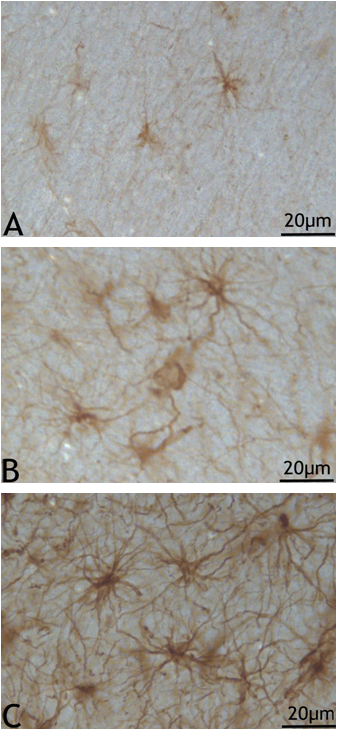

Supplement: S3 Fig — Astrocytes from sham (A), medium (B) and high blast overpressure (C) exposed groups. (TIF) [file pone.0169239.s003.tif]
